# Bone-Anchored Titanium Implants in Patients with Auricular Defects: Three Years and 27 Patients' Experience

**DOI:** 10.1155/2016/9872048

**Published:** 2016-11-09

**Authors:** Emilio Mevio, Luca Facca, Stefano Schettini, Mauro Mullace

**Affiliations:** ^1^Department of Otorhinolaryngology, Fornaroli Hospital, 20013 Magenta, Italy; ^2^F&S Prosthesis Workshop, Ariccia, 00072 Rome, Italy

## Abstract

Different surgical solutions have been proposed for reconstruction of the auricle following loss of the pinna through traumatic injury or neoplastic disease or in patients with congenital defects. Surgical treatment may involve the insertion of an autogenous rib cartilage framework or the use of a porous polymer material inserted into an expanded postauricular flap. Reconstruction with rib cartilage has yielded good results but requires more than one surgical step, and adverse events can occur both at the donor and at the acceptor site; cases of prosthesis rejection have also been described following application of the polymeric prosthesis. The use of a titanium, dowel-retained silicone prosthetic pinna, fixed to the temporal bone, has recently been proposed. This useful surgical approach is indicated particularly after resection of the pinna caused by neoplastic disease or in traumatic auricular injury. Osseointegrated titanium implants used in 27 patients in this study provided them with a safe, reliable, adhesive-free method of anchoring the auricular prostheses. The prostheses allowed recovery of normal physical appearance and all the patients reported that they were completely satisfied with the outcome of the surgical reconstruction. No surgical complications, implant failures, or prosthetic failures were encountered over six months to three years.

## 1. Introduction

Auricular defects characterized by absence of the pinna may be congenital (microtia or anotia) or acquired as a result of infection, cancer surgery, or traumatic injury [1]. Congenital defects may be associated with auditory canal, middle ear, and inner ear malformations [2]. The total or partial pinna mutilation may be a consequence of a car accident, war collateral damage, or chemical burn. Recently an increase in the number of patients presenting with pinna lesions due to chemical assault burns, occurring in the domestic or industrial setting, has been reported [3]. Whatever the cause, absence of the pinna is an important aesthetic problem, with a permanent effect on the patient's quality of life, which can often cause severe psychological distress.

The modern era of pinna reconstruction surgery has its foundations in the techniques proposed by Tanzer, Brent, and Nagata [4–6]. The differing techniques for reconstructive plastic surgery of the auricular defect involve usually the insertion of autogenous rib cartilage framework under the skin. Other surgeons have suggested the employment of prostheses made of synthetic material as a good alternative to autogenous costal cartilage [7, 8]. The reconstructive plastic surgery techniques based on the use of autologous cartilage inserts normally require repeated surgery acts at both implant site and donor site. This creates a significant discomfort both for the pain complained about by the patient and for repeated hospital admissions to the detriment of quality of patient life.

Alternatively, it is possible to use an auricular epithesis. Initially, these epitheses were held in place by adhesives that, however, provided poor results in terms of stability and were often associated with skin irritations and deterioration of the prosthesis caused by the adhesive substances. Also these defects imply a deterioration of patient quality of life.

Currently a superior, innovative surgical technique exists that allows fixation of the ear epitheses by osseointegrated titanium implants (Cochlear Bone Anchored Solutions AB, Sweden). Basically, this approach reflects an evolution of the implants developed for dental prostheses as proposed by Brånemark in 1969, which have been used for over 40 years in the field of odontostomatology and subsequently were proposed for application with osseointegrated craniofacial implants for use with bone-conduction hearing implant solutions [9, 10].

In our study, we describe our experience using these pinna prostheses in 27 patients treated in the last three years. We discuss the indications for auricular epithesis implant, the surgical technique, and outcomes.

## 2. Materials and Methods

### 2.1. Subjects

**Figure 1:**
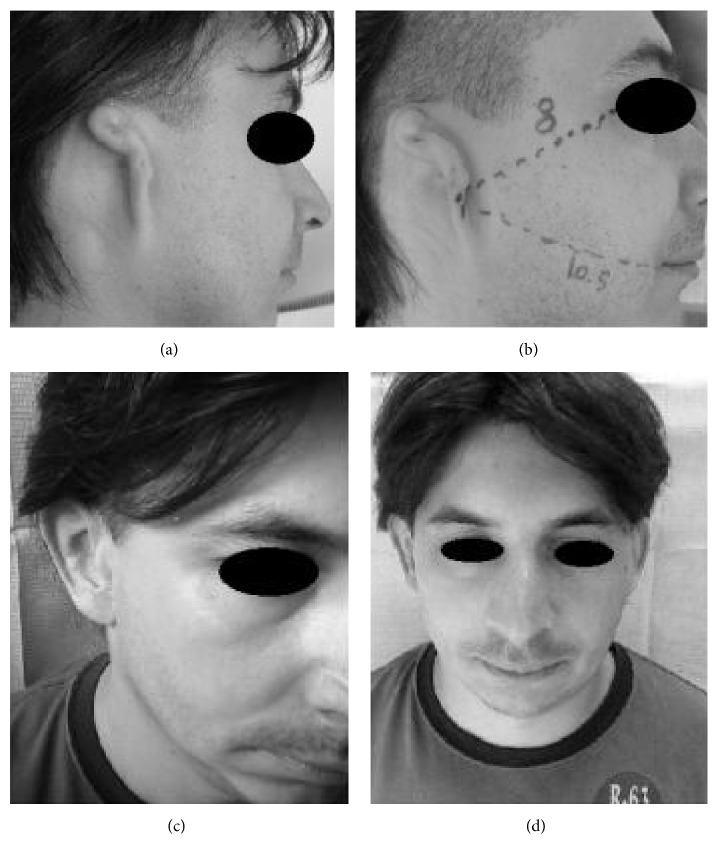
Preoperative image of grade III microtia, right ear (a); (b) shows the technique for determining the proposed location of the external auditory canal; postoperative image in 3/4 right projection (c) and frontal projection (d).

**Figure 2:**
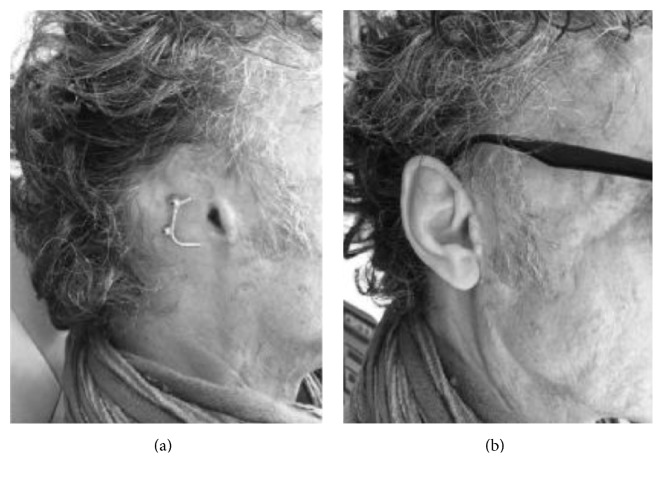
Mutilation of the right auricular pinna caused by a traumatic accident. (b) Epithesis attached to a gold-platinum cylinder-and-bar system.

**Figure 3:**
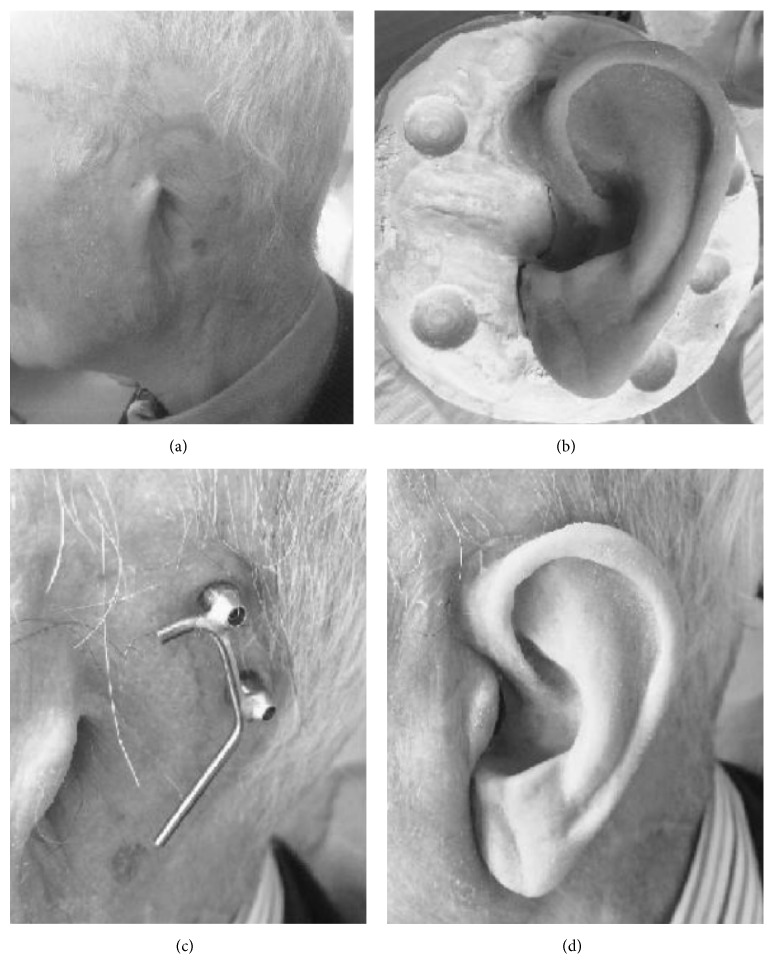
Preoperative image of neoplastic amputation of left ear (a); the silicone epithesis created using a wax pattern (b); gold bar attached to two titanium implants (c); prosthetic pinna in place, clipped onto the gold bar (d).

Our case series comprised 27 patients (24 males and 3 females with a mean age of 33.1 years, range 16–87 years) (Table 1). The etiology was congenital in 17 patients (Figure 1) who were affected by microtia, while eight had posttraumatic mutilation (Figure 2) and two surgical amputation due to neoplasia (Figure 3). Six of the patients with microtia and three of the patients affected by traumatic mutilation had previously undergone plastic reconstructive surgery with rib cartilage grafting and were not satisfied with the results. These patients requested to have their previously reconstructed ear removed and replaced with an epithesis. One patient had previously undergone canaloplasty of the right external auditory meatus. One patient (patient 12) had previously undergone surgery, in a different hospital, using bone-anchored titanium implants but one of the abutments had broken because of a further unlucky trauma.

None of the patients presented with comorbidities.

The follow-up period ranged from six months to three years. After six weeks, all the implants were osseointegrated and a retentive bar was fixed to the abutments.

The patients with microtia were offered the option of undergoing treatment with a bone-conduction hearing implant solution (e.g., BAHA) to concomitantly address their hearing needs in parallel to the auricular rehabilitation with the bone-anchored epitheses. All patients declined the hearing treatment option, preferring to have the option at a later stage instead.

### 2.2. Surgical Procedure

Before the surgical field is prepared and with the patient's face still fully and easily visible, the implant sites should be carefully marked, using methylene blue, down to the bone.

Two implants are normally sufficient for satisfactory prosthesis retention. These are ideally placed approximately 20 mm from the center of the external auditory canal opening or anticipated opening. They are positioned at 8 o'clock and 10:30 on the right side and at 4 o'clock and 1:30 on the left side. In the presence of a complete malformation, the supposed location of the external auditory canal is determined by considering a triangle traced on the contralateral hemiface using the following references: the line between the lateral canthus and the auditory canal, the line between the auditory canal and the labial commissure, and the angle formed by these two lines (Figure 1(b)).

We usually perform one-stage surgery, removing tags and remnants in cases of microtia and performing the necessary subcutaneous tissue reduction.

The one-stage surgical procedure can be used in adults to treat auricular defects involving nonirradiated tissue; the two-stage technique should usually be chosen for paediatric patients and for the treatment of orbital and midface defects and auricular defects in patients with poor bone quality [11].

An incision is made 10 mm behind the anticipated implant site. Dissection is performed down to the periosteum. A cruciate incision is then performed at each implant site. The edges are raised with a raspatory.

Drilling begins using the guide drill with the spacer kept on 3 mm. During drilling, irrigation should be performed. The bottom of the hole is repeatedly checked for bone at the base of the site. If there is adequate bone thickness, drilling continues to a depth of 4 mm. The drill indicator will facilitate correct drill orientation. The next step is to widen the hole to the exact diameter using a 3 or 4 mm drill countersink. Irrigation should always be applied.

At this point, implant installation is performed. The low-speed setting should be used for implant insertion. In compact cortical bone, a torque setting of 40 Ncm is recommended, whereas, in soft bone, a lower torque setting of 20 Ncm should be used.

The self-tapping fixture with the premounted fixture mount is seated inside the plastic ampoule in a titanium cylinder. It is then picked up with the connection to the hand piece, which is placed into the drill hand piece.

The implant is installed without cooling irrigation until the small grooves at the distal end of the implant are well within the canal. When the flange of the implant has seated, the hand piece will automatically stop.

The mount is removed using the* Unigrip *screwdriver and the surgical wrench. The titanium standard abutment is picked up with the abutment holder and placed into the implant. We perform manual tightening, using torque wrench, to 25 Ncm.

The skin is then repositioned over the implants. Holes are punched through the skin exactly over abutments with a biopsy punch. The skin is then sutured. Healing caps are positioned on and attached to the abutments using the Unigrip screwdriver.

Finally a gauze dressing is applied in a figure eight form (i.e., foam dressing, soft silicone wound contact layer, or antiseptic dressing) around the abutments. The healing caps hold the dressing in place during the early postsurgery phase.

### 2.3. Postsurgery

All patients were discharged the day after surgery and were revised for the first dressing after seven days. The patients underwent dressing changes every seven days for a month. Loading the implants occurs six weeks after implant to permit sufficient healing and stabilization of the implants. Following healing and stabilization of the surgical site, the patient was sent to the anaplastology technician who prepared the epithesis, modeling it with reference to the contralateral ear and carefully matching the individual's skin colour (Figures 3(b) and 3(d)). The silicone epithesis was created using a wax pattern. The definitive one has two sides: the inner one is an acrylic plate with clips that allow the attachment to a gold-platinum bar fixed to the abutments and the external one is made of soft silicone. The patients receive two epitheses of different colours: a pale one for winter and a tanned one for summer.

When the process of osseointegration is complete, the prosthesis, which has clips, can be easily and securely attached to, or removed from, the gold-platinum cylinder-and-bar system (Figures 3(c) and 3(d)).

## 3. Results

None of the patients we treated experienced problems related to the implants over six months to three years (osseointegration failure or wound healing problems). All the patients in our series underwent one-stage surgery. Only one patient encountered a relatively exuberant scar in the area of surgical remnants removing. The complication was successfully treated with steroid local infiltration.

The patients were evaluated for their quality of life, during the week before pinna reconstruction with the Short Form Health Survey (SF-12) as a baseline assessment (T0) [12]. Assessment was repeated after three months after implantation (T1). Statistical analysis of changes over time was performed via Pearson Correlations, with a significant change deemed by *p* ≤ 0.05. All our patients expressed satisfaction with their prosthesis. Statistical analysis adopting SF-12 score (Short-Form Health Survey, 12 items) suggests a significant role in quality of life of patients who underwent auricular rehabilitation. All our patients expressed their satisfaction regarding the short hospitalization and reduced invasiveness compared to other alternative therapies. They had no adverse psychological reactions; instead, they were able to resume social relations and usual physical activities.

## 4. Discussion

Absence of the ear, congenital or resulting from trauma or surgery, is a defect that can be resolved through reconstructive plastic surgery. The surgical reconstruction of pinna defects remains a challenging task typically requiring multiple operations with often compromised aesthetic results. This involves the insertion of either an autogenous rib cartilage framework or a prosthesis made from synthetic material into a subcutaneous pocket behind the ear, created through tissue expansion. In the past, if surgical reconstruction was refused, a patient could undergo an alternative rehabilitative approach using an adhesive-retained prosthesis. The reconstruction using rib cartilage has some inherent disadvantages: it requires more than one surgical procedure, the risk of complications is relatively higher both at the implant site (e.g., infections, bleeding, haematoma, necrosis, and skin graft or cartilage graft exposure) and at the donor site (e.g., infections, haematoma, and scarring). Patients are very often dissatisfied with the final outcome because their new ear looks considerably different from the contralateral one and does not meet their expectations [13, 14].

Wellisz proposed the use of* Medpore *prostheses made from porous polymer material inserted in subcutaneous pockets. This procedure is frequently complicated by partial or total rejection and further build-up of scar tissue [7].

The use of adhesive-retained prostheses is also not without its issues, that is, dermatitis resulting from contact with the adhesives, unpredictable reliability of retention, variability of positioning of the prosthesis, and poorer hygiene directly attributable to the tackiness of the adhesive, as well as a decreased life span of the prosthesis resulting in an increased number of device renewals.

Recent studies described the use of osseointegrated auricular prostheses as a good alternative treatment to surgical reconstruction. In this regard, titanium implant systems for bone-anchored implantable hearing solutions have shown us how such prostheses can be attached safely, securely, reproducibly, and without the need for adhesives [11, 15–17]. The procedure discussed in this paper is suitable for patients who are unwilling to undergo plastic reconstructive surgery utilizing rib cartilage, which remains a challenging surgical procedure that involves more than one surgical step and is associated with the risk of complications at the donor site and/or the acceptor site [11].

Similarly, in cases of pinna amputation ensuing following damage to the pinna from chemical burns, the usual techniques of plastic surgery employed hold their inherent difficulties as they involve procedures that require the presence of large areas of intact skin around the lesion (necessary for the preparation of sliding skin flaps or the use of tissue expanders prior to the placement of subcutaneous implants).

Subsequently, osseointegrated implant prostheses are being used increasingly more readily [18]. The use of these implants is also the only possible solution in cosmetic treatment of oncology patients who have previously undergone several surgical procedures and/or radiotherapy [19]. Radiotherapy does not constitute a contraindication for this procedure, although implant loss is relatively higher in irradiated cases than in nonirradiated cases at the site of treatment. Granstrom reported that the adjunctive use of hyperbaric oxygen could ultimately reduce the risk of implant loss [20].

It is important to note the reasonably low cost of the implants and epithesis: typically amounting to a total of less than 4000 Euros. In all previous reports patients express their satisfaction regarding the short hospitalization and reduced invasiveness compared to other alternative therapies.

Complications associated with this surgical technique were rare (10–15% of cases). Local skin infection around the fixture could occur, as could the formation of granulation tissue and keloids. These are complications that can be avoided or resolved using appropriate medication and topical treatments without loss of the fixture [21].

Absolute contraindications for the use of titanium bone implants in prosthetic reconstruction of the auricle are exceptional and may be local or general conditions (i.e., resp., osteitis and terminal illness or the presence of psychological disorders). Contraindications for general anaesthesia need not preclude use of these implants since they can be positioned equally well under local anaesthesia. This surgical technique is contraindicated in patients younger than 14 years of age, whose skull thickness is not sufficient to support the osseointegrated implant. Preoperative evaluation of bone thickness via CT scans should, nevertheless, be a mandatory part of the surgical planning in all the patients [15–17].

As mentioned earlier, indications for one-stage surgery are auricular defects, adult patients, and nonirradiated tissue, while two-stage technique should be used in young patients (14–17 years old) and to treat orbital and midface defects and auricular defects in patients with poor bone quality [11].

Conductive hearing loss due to malformations of the external and middle ear presents in all subjects affected by microtia and can be corrected by combining the placement of titanium implants for auricular rehabilitation with implantation of the fixture and abutment for a BAHA. In this way, both the sensory and the aesthetic problems can be resolved in a single operation. However, it should be pointed out that all the patients in our series refused to undergo BAHA implantation after testing the device prior to surgery; these patients, being well accustomed to hearing on only one side, found the increased auditory perception provided by the BAHA disorienting and irritating.

The pinna epithesis fixed with bone-anchored titanium implants technique is characterized by excellent aesthetic outcome and lasting results. The patients resume social relations and usual physical activities. For example, they are able to use also helmet required for some sport activities and can swim without the problems related to adhesive epitheses.

## Figures and Tables

**Table 1:** 

Patient	Age	Sex	Side	Disease	Previous reconstruction surgery	Follow-up period
1	17	M	Left	Traumatic mutilation	None	3 years
2	25	M	Right	Grade III microtia	Plastic reconstruction	3 years
3	17	M	Right	Grade III microtia	None	3 years
4	34	M	Left	Traumatic mutilation	Plastic reconstruction	3 years
5	44	M	Left	Grade III microtia	Plastic reconstruction	3 years
6	38	M	Right	Grade III microtia	None	2 years
7	16	M	Right	Grade III microtia	None	2 years
8	37	M	Right	Grade III microtia	Plastic reconstruction	2 years
9	19	M	Left	Grade III microtia	None	2 years
10	22	F	Right	Grade III microtia	None	2 years
11	19	M	Right	Grade III microtia	None	2 years
12	26	M	Left	Traumatic mutilation	Vistafix implant	2 years
13	56	M	Right	Traumatic mutilation	None	2 years
14	27	M	Right	Traumatic mutilation	Plastic reconstruction	1 year
15	24	M	Right	Grade III microtia	Canaloplasty	1 year
16	22	F	Right	Traumatic mutilation	None	1 year
17	87	M	Left	Neoplastic amputation	None	1 year
18	28	F	Right	Grade III microtia	Plastic reconstruction	1 year
19	43	M	Left	Grade III microtia	Plastic reconstruction	1 year
20	36	M	Right	Grade III microtia	None	1 year
21	28	M	Bilateral	Burn mutilation	None	1 year
22	28	M	Left	Grade III mutilation	None	1 year
23	63	M	Left	Neoplastic amputation	None	11 months
24	43	M	Left	Grade III microtia	None	11 months
25	39	M	Left	Grade III microtia	None	7 months
26	28	M	Right	Traumatic mutilation	Plastic reconstruction	7 months
27	28	M	Right	Grade III microtia	Plastic reconstruction	6 months
